# Day +60 WT1 assessment on CD34 selected bone marrow better predicts relapse and mortality after allogeneic stem cell transplantation in acute myeloid leukemia patients

**DOI:** 10.3389/fonc.2022.994366

**Published:** 2022-08-31

**Authors:** Patrizia Chiusolo, Elisabetta Metafuni, Gessica Minnella, Sabrina Giammarco, Silvia Bellesi, Monica Rossi, Federica Sorà, Maria Assunta Limongiello, Filippo Frioni, Nicola Piccirillo, Maria Bianchi, Caterina Giovanna Valentini, Luciana Teofili, Simona Sica, Andrea Bacigalupo

**Affiliations:** ^1^ Dipartimento di Diagnostica per Immagini, Radioterapia Oncologica ed Ematologia, Fondazione Policlinico Universitario “A. Gemelli” Istituto di Ricovero e Cura a Carattere Scientifico (IRCCS), Rome, Italy; ^2^ Sezione di Ematologia, Dipartimento di Scienze Radiologiche ed Ematologiche, Università Cattolica del Sacro Cuore, Rome, Italy

**Keywords:** AML, stem cell transplantation, minimal residual disease, stem cell transplant (SCT), minimal residual disease (MRD), WT1, relapse

## Abstract

The aim of this study was to evaluate the role of WT1 expression after allogeneic stem cell transplantation (alloHSCT) in patients with acute myeloid leukemia (AML). We studied WT1 expression in bone marrow cells from 50 patients in complete remission on day +60 after transplant. WT1 was assessed on unfractionated bone marrow mononuclear cells (MNC) and on CD34+ selected cells (CD34+). A ROC curve analysis identified 800 WT1 copies on CD34+ selected cells, as the best cut-off predicting relapse (AUC 0.842, p=0.0006, 85.7% sensitivity and 81.6% specificity) and 100 copies in MNC (AUC 0.819, p=0.007, 83.3% sensitivity and 88.2% specificity). Using the 800 WT1 copy cut off in CD34+ cells, the 2 year cumulative incidence of relapse was 12% vs 38% (p=0.005), and 2 year survival 88% vs 55% (p=0.02). Using the 100 WT1 copy cut off in unfractionated MNC, the 2 year cumulative incidence of relapse 13% vs 44% (p=0.01) and the 2 year survival 88% vs 55% (p=0.08). In a multivariate Cox analysis WT1 expression in CD34 cells proved to highly predictive of relapse (p=0.004); also WT1 expression on unfractionated cells predicted relapse (p=0.03). In conclusion, day-60 WT1 expression after allogeneic HSCT is a significant predictor of relapse, particularly when tested on CD34+ selected bone marrow cells.

## Introduction

Despite advances in treatment and supportive care, the prognosis of adult acute myeloid leukaemia (AML) remains poor with about 40% of young patients and less than 20% of elderly patients surviving in the long term (1). Allogeneic hematopoietic stem cells transplantation (alloHSCT) is the best post-remission treatment for prevention of relapse due to the graft versu leukemia effect (GVL), which is effective regardless of cytogenetic subcategory and minimal residual disease (MRD) status (2, 3).

Patients with positive MRD are considered to be at high risk of recurrence and should receive alloHSCT in first complete remission (CR). AlloHSCT is not indicated in patients with a favourable risk profile (2), whereas patients with favourable risk but persistent MRD are eligible for transplantation (4). The results of alloHSCT compared to autoHSCT and chemotherapy have produced conflicting results in intermediate-risk patients, taking into account molecular markers and MRD status as essential parameters (5–7). In fact, one of the main goals of MRD assessment is to identify, as early as possible the subset of patients at risk of relapse, despite being in CR. This means that these patients can be treated with intensified chemotherapy protocols or transplantation. Unfortunately molecular markers and a leukemia aberrant immunophenotype (LAIP), are not always present in AML patients, making it difficult to establish MRD.

The Wilms’ tumour gene (WT1) was originally identified as a suppressor gene for paediatric Wilms’ kidney cancer. In normal human bone marrow, WT1 is expressed at extremely low levels and is confined to primitive CD34+ cells, but is abnormally expressed in many types of haematological malignancies, making it a molecular marker for leukaemia (8)

The main limitation that prevented the clinical application of this marker for many years was the detection of low transcript levels even in normal haematopoietic cells, suggesting that it could be considered a non-specific marker overexpressed by immature cells. With the introduction of Real Time Quantitative PCR (RQ-PCR) into clinical practice, it became clear that WT1 expression was not only an immaturity marker, but its overexpression was a reliable indicator of the presence of leukemic cells. In particular, WT1 overexpression has been reported in the majority of acute myeloid leukaemia (AML) patients, regardless of the presence of specific fusion transcripts (9).

Several studies have shown that persistence of an abnormal WT1 transcript after chemotherapy, is a strong predictor of subsequent relapse (10). Given the existence of a background WT1 expression in normal bone marrow, qualitative RT-PCR provided conflicting results on the clinical value of this marker (11, 12), whereas RQ-PCR can be used to assess different levels of WT1 transcripts in AML cells, normal hematopoietic cells and normal bone marrow cells regenerating after chemotherapy (13, 14). Therefore, longitudinal RQ-PCR analysis of the amount of WT1 transcript may be clinically relevant for monitoring AML.

In a retrospective study on a cohort of patients submitted to alloHSCT we demonstrated that WT1 expression on bone marrow mononuclear cells (MNCs) is predictive of leukemic relapse, and can be used to initiate immunotherapy with donor lymphocyte infusion using as cut off < 100 WT1 copies normalised to 10^4^ Abelson copies (ABL) (14). We found that patients with WT1 copies >100 had a 54% probability of relapse whilst patients with copies <100 had a 16% probability of relapse.

In a more recent study from our group, in addition to confirming the data, we showed that by administering immunotherapy (IT) in two different groups defined by the expression levels of Wt1 copies >180(WT1-180) and Wt1 copies >100(WT1-100) the cumulative incidence of recurrence was 76% in the WT1-180 group compared to 29% in the WT1-100 group, i.e. a significant improvement in MRD positive disease free survival of 23% compared to 74% (15). Therefore, WT1 is a sensitive marker of leukemic relapse, and predictive therapy is feasible by defining an expression level >100 copies as a cut off. Several studies have confirmed that WT1 expression before and/or after allogeneic transplantation predicts leukemia relapse (16–19).

The aim of the present study is to further increase the predictive role of WT1 expression by evaluating selected CD34\+ cells, isolated from bone marrow on day +60 after allo-HSCT.

## Methods

### Study population

AML patients undergoing alloHSCT at Fondazione Policlinico A. Gemelli IRCCS from June 2018 to July 2020 were prospectively investigated. Healthy bone marrow donors were included as controls. The study was approved by the local Ethic Committee (Prot.4065/21 April 28, 2021).

### Patient, donor, and graft data

Patients’ variables included demographics, diagnosis and date of diagnosis, date of transplant, disease status (complete remission or not), disease risk index (DRI), European Leukemia Net (ELN) risk, hematopoietic cell transplantation comorbidity index (HCT-CI), date of acute or chronic GVHD (aGVHD and cGVHD), date of relapse, date of death, or last follow-up. Donor variables included HLA match, age, and gender.

### Cell samples and quantitative assessment of *WT1* expression

WT1 expression was evaluated on both MNCs and CD34+ cell samples. Mononuclear cells were separated on a Ficoll-Hypaque (Lymphophlot; Bio-RAD Medical Diagnostics GmBH, Dreireich, Germany) density gradient. Total RNA was extracted using Trizol (Invitrogen, Life Technologies, CA), following the manufacturer’s instructions. CD34+ cells were isolated from MNCs by immunomagnetic method (Miltenyi, Biotech, Bergish Gladbach, Germany).

All analysis were performed in triplicate. For quantitative assessment of *WT1* mRNA, a calibration curve with a plasmid containing the *WT1* target sequence was used (ProfileQuant WT1 Kit, European Leukemia Net, Ipsogen, France). The *WT1* ProfileQuant kit includes specific plasmids and primers and probe mixes for *WT1* and Abl. These components have been validated together in the context of a collaborative study led by a group of experts from the European LeukemiaNet consortium (10). RQ-PCR reactions and fluorescence measurements were made on the RotorGene3000 (Corbett Life Science, Sydney, Australia). The *WT1* mRNA levels of expression were normalized with respect to the number of Abl transcripts and expressed as *WT1* copy numbers/10^4^ copies of Abl.

For each patient, a bone marrow sample was collected on day +60 after transplantation. WT1 copy number data normalised for 10^4^ Abl copies was obtained on selected CD34+ cells in 45 patients and on whole bone marrow mononuclear cells in 40 patients.

In addition, two control groups of healthy bone marrow donors were enrolled and an aliquot of the graft was used for WT1 determination. In one donor group of 42 subjects, WT1 was evaluated on selected CD34+ cells, while in the other group of 18 healthy donors WT1 was determined on whole bone marrow mononuclear cells.

### Statistical analysis

The continuous numeric variable WT1 was compared between groups using the Mann-Whitney and Kruskal-Wallis tests. Using the Receiver Operating Characteristics (ROC) curve, the cut-off of the continuous variable WT1 was defined in relation to the relapse outcome, and for this cut-off the percentage of sensitivity and specificity was reported, as well as the area under the curve (AUC) of the ROC and its relative 95% confidence interval. The continuous variable WT1 was then transformed into a categorical variable as a function of the cut-off defined by the ROC curve. Categorial variables were compared by Chi square and Fisher exact test between patients with and without relapse. Univariate and multivariate analysis were performed with the Cox regression model for relapse and survival with the following variables: patients age, donor HLA matching, intensity of the conditioning regimen (myeloablative, reduced intensity), adverse karyotype (yes/no), adverse ELN risk (yes/no), remission status at transplants (yes/no), stem cell source (peripheral blood/(bone marrow), and WT1 expression in CD34+ cells, or WT1 expression in unfractionated BM cells. Cumulative relapse incidence curves were compared by Grays test.

Kaplan Meier curves were drawn for survival and compared with the log-rank test. The statistical analysis was carried out with the NCSS19 software.

## Results

In total 50 AML patients and 60 donors were included in the study. Patients and transplant characteristics are shown in **Table 1**. The median age was 56 years (25–69). The ELN risk groups were as follow: favourable (n=10), intermediate (n=27), adverse (n=13).

**Table 1:** Patient’s characteristics.

Patients	50
**Age, median (range)**	56 ys (25-69)
**Gender, F/M**	24/26
**ELN Risk** Favourable Intermediate unfavourable	10 (20%) 27 (54%) 13 (26%)
**Molecular Markers** NPM FLT3 t(8;21) Inv(16) c-kit	17 (34%) 14 (28%) 1 (2%) 4 (8%) 1 (2%)
**Time from diagnosis to transplant, median (range)**	186 days (50-935)
**Disease status at transplant** 1 CR 2 CR PR Relapsed/refractory	30 (60%) 5 (10%) 4 (8%) 11 (22%)
**Donor match** Sibling Haplo MUD MMUD	8 (16%) 16 (32%) 13 (26%) 13 (26%)
**HCT-CI, median (range)**	3 (0-6)
**Conditioning regimen** MA RIC	22 (44%) 28 (56%)
**GvHD prophylaxis** CSA+MTX+ATG CSA+Cy CSA+MMF+Cy	7 (14%) 1 (2%) 42 (84%)
**CD34+, median (range)**	5.75 x10^6^/Kg (0.1-10.8)
**Stem cells source** PB BM CB	30 (60%) 16 (32%) 4 (8%)
**Donor** Related Unrelated	24 (48%) 26 (52%)

ELN = European Leukemia Net risk; HSCT = hematopoietic stem cell transplantation; CR =complete remission; PR =partial remission; MA= myeloablative conditioning; RIC= reduced intensity conditioning; MUD = matched unrelated donor; MMUD= mismatched UD; Haplo= haploidentical donor; sibling= HLA matched sibling; ATG = antithymocyte globulin; PB = peripheral blood; BM= bone marrow; CB= cord blood; aGvHD = acute graft-versus-host disease; CD34+ = selected CD34+ cells on bone marrow samples; MNC= total mononucleated cells in bone marrow.

CSA = cyclosporin; MMF= mycophenolate; CY= cyclophosphamide

Seventeen patients (34%) developed aGvHD after a median of 34 days (range 16-90). Grading was as follows: grade I in 12 patients (70.6%), grade II in 4 patients (23.5%) and grade III in 1 patient (5.9%). Chronic GvHD was diagnosed in 13 (28.3%) of the 46 patients with a follow-up of more than 100 days. Grading was as follows: mild in 9 patients (69.2%) and moderate in 4 patients (30.8%).

Eleven patients (22%) relapsed after a median of 120 days after transplantation (range 73-582), while the others maintained a complete remission at the follow-up time of July 2021. At the same follow-up time, 40 patients (80%) were alive with a median survival of 435 days (range 84-861), while 10 patients (20%) died after a median time of 186 days (range 96-334). The causes of death were as follows: transplant-related mortality in 3 patients (6%) and disease recurrence in 7 patients (14%).

### WT1 expression in patients and controls

The expression of WT1 was assessed at day + 60 in 50 AML patients: in 40 patients both CD34+ cells and MNCs were evaluated, while in further 10 patients, WT1 was evaluated only in CD34+ cells (5 patients) or MNCs (5 patients). Moreover, 42 CD34+ cell samples and 18 MNCs samples from healthy bone marrow donors were used as controls.

We first compared WT1 expression in patients and controls. No difference was seen between patients (49.7 copies, 95%C.I 29.6-67.3) and controls (43.2 copies, 95%CI 17.1-59.5) looking at WT1 expression on total bone marrow MNC (p=0.2). On the contrary a statistically significant difference was observed between the median WT1 levels on selected bone marrow CD34+ cells between the two groups: 406.5 copies for patients (95%CI 342.8-634.6) and 252.3 copies in controls (95%CI 188.9-314.2) (p=0.0007).

**Figure 1:**
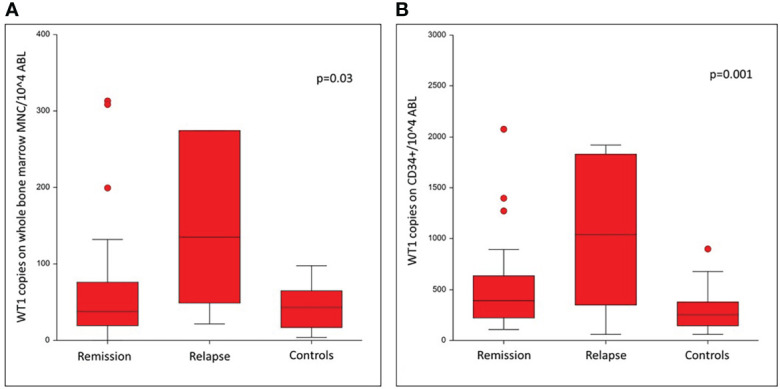
Comparison of WT1 levels on day +60 after allogeneic HSCT for patients in remission, patients relapsed and in controls.

WT1 expression on total bone marrow MNC was significantly different in patients who remained in remission (37.9 copies - 95%CI 25.5-60.2), as compared to patients who relapsed (135.3 copies 95%CI 21.4-1072.8) and to controls (43.2 copies, 95% CI 17.1-59.5) (p=0.03) (**Figure 1A**). In CD34 + cells the median WT1 copy number was 389.2 copies for patients who remained in remission (95% CI 246.3-472.2), and 1129.1 copies for patients who relapsed (95% CI 58.8-1918.2) and 252.3 for controls (95% CI 188.9-314.2) (p=0.001) (**Figure 1B**).

### ROC curve for WT1 with relapse outcome

**Figure 2:**
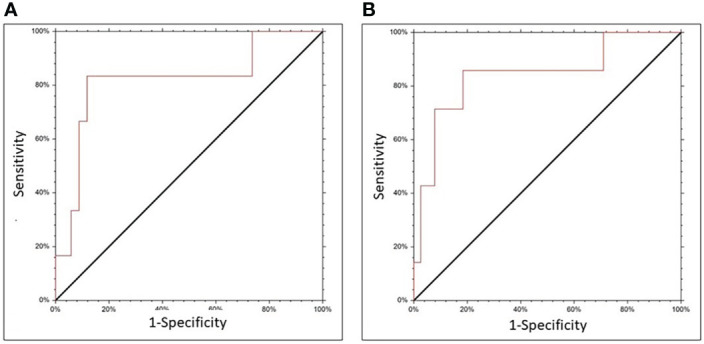
ROC curve for WT1 expression on CD34 selected cells **(A)** or unfractionated BM cells **(B)**, and relapse.

The ROC curve was then used to define a threshold of MRD of WT1 for the relapse outcome. For WT1 level on whole bone marrow MNC the AUC was 0.819 (CI 95% 0.426-0.952). The selected cut-off was 100 copies, with a sensitivity of 83.3% and a specificity of 88.2%. (p=0.007, **Figure 2A**). For the WT1 level determined on selected bone marrow CD34+ cells, the AUC was 0.842 (95% CI 0.508-0.956). The selected WT1 cut-off was 800 copies, with a sensitivity of 85.7% and a specificity of 81.6% (p=0.0006, **Figure 2B**). Using the cut-offs identified with the ROC curve, the continuous WT1 levels variable was transformed into a categorical variable.

### Univariate analysis

**Figure 3:**
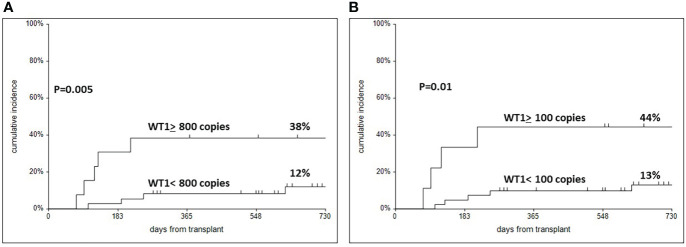
Cumulative incidence of relapse in patients according to WT1 expression in CD34 selected cells **(A)** with a cut off of 800, and in unmanupulated mononuclear cells with a cut off of 100 **(B)**.

Comparing patients who later relapsed, with patients in continuous remission (**Table 2**), significant difference were found in the proportion of patients with an adverse Karyotype (p= 0.02), and in the proportion of patients with a high WT1 day+60 expression, both on CD34+ cells as well as on unfractionated BM cells (**Table 2**). The cumulative incidence of relapse is shown in **Figure 3**: when using the 800 WT1 copies cut off, on CD34+ cells, the 2 year cumulative incidence of relapse was 12% vs 38% (p=0.005) (**Figure 3A**); when using the 100 WT1 copies cut off on unfractionated BM cells, the 2 year cumulative incidence of relapse was 13% vs 44% (p=0.01) (**Figure 3B**).

**Table 2:** Characteristics of patients who subsequently did or did not relapse.

	RELAPSE	RELAPSE	P
	Yes	No	
Recipients age	59 (42-66)	53 (49-57)	0.3
Adverse karyotype	44%	14%	0.02
Adverse ELN	44%	28%	0.2
CR at transplant	55%	73%	0.3
Myeloablative conditioning	84%	81%	0.8
HLA matched donor	11%	35%	0.08
ATG in the conditioning	11%	15%	0.8
Stem cell source PB	81%	74%	0.6
HCT-CI,median (range)	3(2-4)	3 (2-3)	0.4
Acute GvHD II-IV	18%	8%	0.3
WT1 >800 copies *	60%	20%	0.01
WT1 >100 copies **	57%	15%	0.01

HCT-CI =hemopoietic stemc cell transplant- comorbidity index. ATG = anti-thymocyte globulin. * on CD34+ cells; ** on unfractionated BM cells.

### Cox analysis on relapse

In univariate analysis, significant predictors, were WT1 expression on CD34+ cells and unfractionated cells, as well as an adverse karyotype and adverse ELN risk group. In multivariate analysis WT1 expression was entered either from CD34+ cells or from unfractionated BM cells: both were predictive of relapse (**Table 3**).

**Table 3:** Univariate and multivariate Cox analysis on relapse.

	UNIVARIATE			MULTIVARIATE			MULTIVARIATE		
Variable	RR	95%CI	P	RR	95%CI	P	RR	95%CI	P
WT1>800*	5.6	1.5-20	.008	8.5	1.9-37	.004			
WT1 >100**	8.8	1.6-48	.01				6.8	1.1-39	.03
Adverse karyotype	7.2	2.1-23	.001	8.6	0.3-48	.1	6.4	0.1-22	.1
Adverse ELN	3.9	1.2-12	.02	1.3	0.6-29	.8	2.1	.1-18	.6
Age >60 years	1.4	0.3-5.4	.6	–	–	–	–	–	–
CR at transplant	0.4	0.1-1.2	.09	0.8	0.2-3.3	.8	0.3	0.1-8	.7
HLA matched don	2.1	0.6-7	.2	–	–	–	–	–	–
MA regimen	0.5	0.1-2	.5	–	–	–	–	–	–
PB vs BM	1.0	0.2-4	.9	–	–	–	–	–	–

as in **Tables 1** and **2**

### WT1 expression and survival

**Figure 4:**
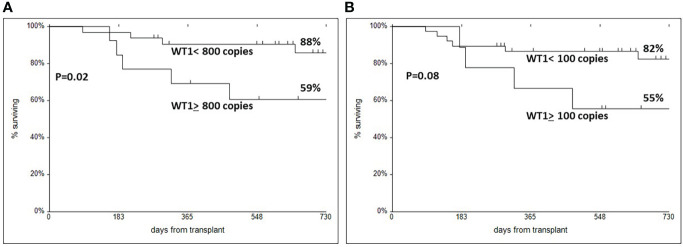
Actuarial two year survival in patients with a cut off of WT1 800 copies for CD34 selected BM cells **(A)**, or WT1 100 copies for unfractionated BM cells **(B)**.

The two year survival of patients stratified according to WT1 expression on CD34+ cells was 88% vs 59% (p=0.02) (**Figure 4A**); the survival of patients stratified according to WT1 expression on unfractionated BM cells was 82% vs 55% (p=0.08) (**Figure 4B**). DFS was also predicted by WT1 expression on CD34+ cells (79% vs 61%, p=0.03, with the 800 copy cut off), and also on unfractionated BM cells (85% vs 56%, p=0.01, with the 100 copy cut off).

In a Cox multivariate analysis on survival, age >60 years was a significant predictor (p=0.03) together with WT1 copy number over 800 for CD34+ cells (RR 18.1, p=0.05) and less so for WT1 copy number over 100 for unfractionated BM cells (RR 4.2, p=0.09). Similarly in a Cox model for disease free survival WT1 expression on CD34+ cells was a better predictor of failure (RR 5.8, p=0.01) as compared to WT1 expression on unfractionated BM cells (RR 7.4, p=0.04), together with age >60 years (RR 8.4, p=0.02).

## Discussion

The quantification of MRD is considered a powerful, independent predictive factor after HSCT. Monitoring leukemia-specific gene mutation by PCR or LAIP represents the gold standard to stratify patients on the basis of the risk to relapse. Unfortunately, more than 50% of AML cases lack specific genes and 10-30% of them lack LAIP. The National Cancer Institute’s second workshop on relapse after HSCT (20) identified several topics for the prevention of leukemia relapse, including “detection and preventive therapy of impending relapse”. Three papers addressed the issue of WT1 as a marker of MRD in AML after transplantation (21–23) and were able to identify a predictive association between WT1 levels and relapse. Rossi et al. found that high WT1 levels at 1 month from the transplant significantly impacted on DFS (.p = 0.010) and had a higher predictive value than WT1 levels on days +90 (21). Israyelyan et al. focused on the period after alloHSCT for predicting relapse onset using WT1 overexpression and looked at WT1 levels on peripheral blood cells and determined a cut-off level that would identify patients at risk of hematological relapse (22). Both cut-off levels of 50 and 20 reproduced high specificity and sensitivity. The WT1/c-ABL transcript ratio of 50 or above demonstrated 100% specificity and 75% sensitivity predicting relapse with an observed average of 29 days, while a lower ratio of 20 or above had lower specificity, but higher sensitivity (84.8% and 87.5%, respectively) and identified more patients who had an hematological relapse, at earlier times, providing an earlier warning with actual average lead time of 49 days. Using the ratio of 20 (HR 58.16, p<0.0001) WT1, together with high risk disease (HR 3.27, p=0.02) and donor age above 34 years (HR 5.12, p=0.01), are listed as predictor variables for relapse occurrence. Among these, multivariate analysis confirmed only WT1 ratio of 20 as associated with decreased time to relapse (22). Yoon et al. (23) examined WT1 transcription levels in bone marrow MNC one month after transplantation in patients with refractory anemia with excess blasts demonstrating that a cut-off level of 154 copies at 1 month was predictive of leukemia relapse. In this study, 47% of patients who exceeded this cut-off level, versus 7% of patients who did not reach 154 copies, relapsed. Multivariate analysis confirmed high WT1 expression (HR 9.94, p=0.002) and poor karyotype before transplant (HR 3.52, p=0.05) as predictive variables for subsequent relapse. A further study showed that low WT1 levels after transplantation were associated with higher and longer-lasting frequencies of WT1-specific cytotoxic T cells (CTLs) in long-term survivors (24). High WT1 levels in autologous peripheral blood apheresis were also shown to predict relapse in AML patients (25).

Pozzi et al. also confirmed that AML patients in CR before transplant and with a median expression of WT1 >100/10^4^ ABL after transplant had a higher relapse risk (53% vs 26%) and a lower 5-year survival (36% vs 62%) when compared with patients who had less than this cutoff (14). In multivariate analysis predicting factors for relapse were: disease phase at transplant (RR 2.3, p=0.002), pre-transplant WT1 level (RR 2.2, p=0.01) and post-transplant WT1 level (RR 4.5, p=0.0001) determined on bone marrow samples.

In a more recent study the same group (15) examined the efficacy of IT (consisting of cyclosporine interruption and infusion of donor lymphocytes) triggered at different levels of MRD expression: patients treated at a cut-off level WT1 expression in marrow cells of 100 copies had a significantly lower risk of progressing to hematological relapse than patients treated at a higher cut-off level (180 copies) demonstrating that the greater efficacy of IT in WT1-100 patients is due to the fact that the intervention occurred with a lower disease burden. The greater effect of IT in WT1-100 patients was also demonstrated by the higher percentage of patients achieving molecular remission: 96% compared to 35% of WT1-180 patients (15).

The goal of our study was to evaluate a greater predictivity of WT1 expression in CD34 + cells as compared to the expression levels on unfractionated MNCs after alloHSCT in AML patients. We evaluated WT1 expression levels in selected bone marrow CD34 + cells of 50 patients at day 60 post HCST.

Using the ROC curve it was possible to define a cut off equal to 800 copies in CD34 + selected from MNC on bone marrow and a cut off equal to 100 copies on unfractionated mononuclear cells from bone marrow, confirming the results of Pozzi et al. (14).

In particular, in a multivariate Cox model, patients with WT1 ≥ 800 copies on selected CD34 + bone marrow cells, had a 8.5-fold higher risk of relapse, as compared to patients with WT1 <800 copies. The predictive value of WT1 expression over 100 copies, on unfractionated bone marrow mononuclear cells, was predictive of relapse (6.8-fold greater risk), but with less statistical power (p=0.03 as compared to p=0.004 for CD34+ cells). WT1 expression on CD34+ cells was also predictive of survival in a multivariate analysis (p=0.05) and disease free survival (p=0.01) together with patients age > 60 years (p=0.03). The predictive role of WT1 expression on unfractionated BM cells was less significant for survival (p=0.09) and disease free survival (p=0.04). So the expression of WT1 on CD34+ cells appeared to provide a higher predictive value in the multivariate Cox model.

In conclusion, the expression of WT1 on CD34 cells selected on day +60 after allogeneic transplantation, is greater as compared to WT1 expression on unfractionated bone marrow MNC, and provides a predictive assay for leukemic recurrence after alloSCT. We would favor CD34 selected cells to assess MRD on day +60 after transplant, and thus predict relapse, in particular in patients not expressing LAIP or molecular markers suitable for MRD monitoring after transplant.

## Data availability statement

The data analyzed in this study is subject to the following licenses/restrictions: The dataset will be available upon request to the corresponding author. Requests to access these datasets should be directed to patrizia.chiusolo@unicatt.it.

## Ethics statement

The studies involving human participants were reviewed and approved by Comitato Etico-Fondazione Policlinico Universitario Agostino Gemelli IRCCS. The patients/participants provided their written informed consent to participate in this study.

## Author contributions

PC and AB designed research. GM, SG, and MR performed research and analyzed data. EM, SG, FS, ML, FF, NP, MB, CG, LT, and SS treated the patients. PC, EM, and AB wrote the paper and all co-authors reviewed the manuscript. All authors contributed to the article and approved the submitted version.

## Funding

This study was supported in part by Associazione Italiana Ricerca contro il Cancro (AIRC) Milano (AIRC 2017 IG 20132).

## Conflict of interest

The authors declare that the research was conducted in the absence of any commercial or financial relationships that could be construed as a potential conflict of interest.

## Publisher’s note

All claims expressed in this article are solely those of the authors and do not necessarily represent those of their affiliated organizations, or those of the publisher, the editors and the reviewers. Any product that may be evaluated in this article, or claim that may be made by its manufacturer, is not guaranteed or endorsed by the publisher.
